# Processing and secretion of guanylate binding protein‐1 depend on inflammatory caspase activity

**DOI:** 10.1111/jcmm.13116

**Published:** 2017-03-08

**Authors:** Elisabeth Naschberger, Walter Geißdörfer, Christian Bogdan, Philipp Tripal, Elisabeth Kremmer, Michael Stürzl, Nathalie Britzen‐Laurent

**Affiliations:** ^1^ Division of Molecular and Experimental Surgery Department of Surgery Friedrich‐Alexander‐Universität (FAU) Erlangen‐Nürnberg and Universitätsklinikum Erlangen Translational Research Center Erlangen Germany; ^2^ Mikrobiologisches Institut – Klinische Mikrobiologie, Immunologie und Hygiene Friedrich‐Alexander‐Universität (FAU) Erlangen‐Nürnberg and Universitätsklinikum Erlangen Erlangen Germany; ^3^ Institute of Molecular Immunology Helmholtz Zentrum Munich German Research Center for Environmental Health Munich Germany; ^4^ Present address: Optical Imaging Center Erlangen (OICE) Friedrich‐Alexander‐Universität (FAU) Erlangen‐Nürnberg Erlangen Germany

**Keywords:** guanylate binding protein, interferon, GTPase, Secretion, Inflammation, caspase‐1, caspase‐5, endothelial cells, HUVEC

## Abstract

Human guanylate binding protein‐1 (GBP‐1) belongs to the family of large GTPases. The expression of GBP‐1 is inducible by inflammatory cytokines, and the protein is involved in inflammatory processes and host defence against cellular pathogens. GBP‐1 is the first GTPase which was described to be secreted by eukaryotic cells. Here, we report that precipitation of GBP‐1 with GMP‐agarose from cell culture supernatants co‐purified a 47‐kD fragment of GBP‐1 (p47‐GBP‐1) in addition to the 67‐kD full‐length form. MALDI‐TOF sequencing revealed that p47‐GBP‐1 corresponds to the C‐terminal helical part of GBP‐1 and lacks most of the globular GTPase domain. *In silico* analyses of protease target sites, together with cleavage experiments *in vitro* and *in vivo*, showed that p67‐GBP‐1 is cleaved by the inflammatory caspases 1 and 5, leading to the formation of p47‐GBP‐1. Furthermore, the secretion of p47‐GBP‐1 was found to occur *via* a non‐classical secretion pathway and to be dependent on caspase‐1 activity but independent of inflammasome activation. Finally, we showed that p47‐GBP‐1 represents the predominant form of secreted GBP‐1, both in cell culture supernatants and, *in vivo,* in the cerebrospinal fluid of patients with bacterial meningitis, indicating that it may represent the biologically active form of extracellular GBP‐1. These findings confirm the involvement of caspase‐1 in non‐classical secretion mechanisms and open novel perspectives for the extracellular function of secreted GBP‐1.

## Introduction

The 65‐ to 73‐kD guanylate binding proteins (p65‐GBPs) belong to the major interferon (IFN)‐γ‐induced GTPases. Large GTPases of the GBP family are involved in inflammatory processes 1, 2, 3, 4, 5, 6. In addition, several human and mouse GBPs have been shown to be involved in the response against intracellular pathogens including viruses as well as bacterial, mycobacterial and parasitic infectious agents 6, 7, 8, 9, 10, 11, 12, 13, 14, 15, 16. The human GBP family consists of seven members, which exhibit a high degree of homology among each other 17, 18. GBP‐1 is the best characterized member of the family and comprises two structural domains: an N‐terminal globular domain with GTPase activity and a C‐terminal α‐helical domain 19, 20. GBP‐1 hydrolyses GTP with a high turnover rate to GDP or GMP and orthophosphates 21, 22. During the GTPase cycle, GBP‐1 undergoes nucleotide‐dependent oligomerization 19.

GBP‐1 is expressed in many different cell types under inflammatory conditions *in vitro*, for instance after cytokine treatment (*e.g*. IFN‐γ)*,* but it is preferentially associated with endothelial cells *in vivo* 1. Expression analyses of all members of the human GBP family in endothelial cells revealed that GBP‐1, GBP‐2 and GBP‐3 are induced by IFN‐γ, interleukin (IL)‐1β and tumour necrosis factor (TNF)‐α, whereas GBP‐4 and GBP‐5 are only induced by IFN‐γ 23, 24, 25. Expression of GBP‐6 and GBP‐7 was not detectable upon stimulation with IFNs, IL‐1β or TNF‐α in endothelial cells 18, 23.

Analyses of the functions of GBP‐1 showed that the protein mediates the inhibition of proliferation, spreading, migration and invasion of primary endothelial cells in response to inflammatory cytokines 6, 23, 24, 26. Furthermore, GBP‐1 was shown to be an independent prognostic factor in colorectal carcinoma (CRC), associated with a prolonged survival and the presence of an angiostatic micromilieu characterized by a quiescent mature vasculature under the control of SPARCL1 27, 28. In CRC cells, GBP‐1 was found to exert antitumorigenic effects both *in vitro* and *in vivo* 29. In addition, we previously reported that GBP‐1 can be detected in body fluids during infectious and inflammatory diseases including bacterial meningitis, systemic lupus erythematosus, rheumatoid arthritis and systemic sclerosis 30, 31. Interestingly, we could show that GBP‐1 is actively and selectively secreted from endothelial cells in the absence of leader peptide 30.

IL‐1β is the prototype of a non‐classically secreted protein 32, 33. Secretion of IL‐1β and other leaderless proteins has been shown to depend on the enzymatic activity of caspase‐1 34. In the case of IL‐1β, caspase‐1 is also critical for processing of the inactive pro‐form to mature IL‐1β 32, 33, 35, 36.

In this study, we analysed the release of GBP‐1 from endothelial cells in more detail. A 47‐kD protein fragment, which corresponds to the C‐terminal part of GBP‐1, was co‐precipitated together with the full‐length 67‐kD form of the protein from the cell culture supernatants of primary endothelial cells. As GBP‐1 harbours a potential inflammatory caspase cleavage site, we investigated whether caspase‐1/‐4/‐5 activity may be involved in the generation and the secretion of the 47‐kD GBP‐1 fragment. In addition, we assessed the quantitative relationship between the 67‐kD and the 47‐kD extracellular forms of the protein, as well as the mode of secretion and the presence of p47‐GBP‐1 *in vivo*.

## Materials and methods

### Plasmids

The plasmids pMCV1.4 and pMCV2.2 (Mologen, Berlin, Germany) were used for transient and stable cDNA expression, respectively. Each cDNA‐encoded protein was fused with a Flag tag (Flag or F) at the N‐terminus. pMCV‐Flag‐GBP‐1, pMCV‐Flag‐GFP and pMCV‐Flag‐GBP‐1‐D184N were described previously 30. The pMCV‐Flag‐GBP‐1‐D192E vector was generated with QuikChange site‐directed mutagenesis (Stratagene, La Jolla, CA, USA) performed with pMCV‐Flag‐GBP‐1 as a template. The pMCV‐Ost‐Flag‐GBP‐1 plasmids were obtained by PCR‐cloning of the Osteonectin signal peptide (Ost: N‐MRAWIFFLLCLAGRALA/AP‐C) sequence in front of the Flag tag, together with a glycine linker consisting of a stretch of nine consecutive glycine codons. The plasmids pMCV1.4‐Flag‐GBP‐1‐globular domain and pMCV1.4‐Flag‐GBP‐1‐helical domain consisted of the Flag tag fused to the amino acids (aa) 1‐290 (glob) and aa 291‐592 of human GBP‐1 (hel), respectively. The integrity of all cDNAs was confirmed by sequencing.

### Recombinant human GBP‐1

The generation of recombinant human GBP‐1 was described previously 37.

### Cell culture

Human umbilical vein endothelial cells (HUVECs) were purchased from Lonza (Verviers, Belgium) or Promocell (Heidelberg, Germany) and used between passage numbers 4 and 9. A 1:4 split ratio was defined as one passage. Cells purchased from Lonza were grown in endothelial cell basal medium (EBM‐2‐MV; Lonza), supplemented with 5% (v/v) foetal calf serum (FCS) (EBM‐2‐full medium) and propagated in cell culture flasks (Nunc, Wiesbaden, Germany) coated with 1.5% (w/v) bovine skin gelatin, type B (Sigma‐Aldrich, Munich, Germany) in PBS. Cells purchased from Promocell were grown in endothelial cell basal medium (ECGM, Promocell). The monocytic THP‐1 cell line (ATCC^®^‐TIB‐202) was cultivated in RPMI supplemented with 10% (v/v) foetal calf serum (FCS). Cells were routinely tested with a mycoplasma detection kit (Lonza) and were found to be not infected. IFN‐γ stimulation (100 U/ml; Roche) was carried out in low‐FCS medium (0.5% FCS, without supplements). Z‐VAD‐fmk and Z‐YVAD‐fmk (both from R&D systems, Wiesbaden, Germany) were added to the cultures 6 hrs after the stimulation with IFN‐γ. Z‐VAD‐fmk and Z‐YVAD‐fmk were dissolved in dimethylsulphoxide (DMSO). Glyburide [5‐chloro‐N‐(4‐(cyclohexylureidosulfonyl)phenethyl)‐2‐methoxybenzamide] and monensin A sodium salt were purchased from Sigma‐Aldrich and dissolved in DMSO or methanol, respectively. PMA (Phorbol 12‐myristate 13‐acetate) was purchased from Sigma‐Aldrich and dissolved in DMSO. LPS (Lipopolysaccharide) and ATP (Adenosine triphosphate) were purchased from Sigma‐Aldrich. Final concentrations of organic solvents in culture medium were always kept below 0.5% to avoid toxicity.

### Transfection

HUVEC were seeded into a 25‐cm² flask or a 10‐cm dish at a density of 12,000 cells/cm^2^. After 24 hrs, a mixture of plasmid and Superfect (Qiagen) [1:10 (m/v)] was diluted in basal culture medium and pre‐incubated for 10 min at room temperature before being added to the cells kept in EBM‐2‐MV medium. Cells were washed after 2 hrs with PBS, and fresh medium, optionally supplemented with glyburide, monensin or Z‐YVAD‐fmk, was added. Cells were further cultured for 28–44 hrs before cell culture supernatants (SN) were harvested and cell lysates prepared.

Hela cells were seeded with 1.2 × 10^5^ cells per well in six‐well plates and transfected using 5 μg plasmid by the calcium phosphate method as previously described 38. Medium was renewed 8 hrs after transfection, and cells were harvested 48 hrs after transfection.

### GMP‐agarose and immunoprecipitation

IFN‐γ‐stimulated HUVECs were resuspended in CSK buffer [1% Triton X‐100, 10 mM PIPES (pH 6.8), 300 mM sucrose, 100 mM KCl, 2.5 mM MgCl_2_, one protease inhibitor tablet without EDTA (Roche) per 10 ml buffer] for the generation of cell lysates as described previously 39. Supernatants from IFN‐γ‐stimulated HUVECs were supplemented with protease inhibitor without EDTA, centrifuged (5 min; 1000 × *g*) and concentrated *via* Vivaspin (MWCO 10 kD) (Sartorius, Goettingen, Germany). Lysates and supernatants were incubated in a rotator overnight with 100 μl GMP‐agarose (Sigma‐Aldrich) at 4°C. The agarose beads were centrifuged (1700 × *g*; 5 min, 4°C), washed with 20 mM Tris/HCl, 5 mM MgCl_2_, 15 mM NaCl and 1 mM DTT and boiled for 5 min in 2× Laemmli buffer with β‐mercaptoethanol to eluate the proteins. Immunoprecipitation using anti‐Flag antibodies or polyclonal rabbit anti‐GBP‐1 antibodies was carried out as described previously 30.

### Acetone precipitation

Cell culture supernatants of HUVECs (300 μl) and CSF samples (400 μl) were precipitated with 4 volumes of pre‐chilled acetone and incubated overnight at −20°C followed by centrifugation for 10 min at 15,000 × *g* and 4°C. Protein pellets were re‐suspended in 2× Laemmli buffer and boiled for 10 min.

### Western blot analysis

Western blotting was performed as described previously 23. The following primary antibodies were used: monoclonal rat anti‐human GBP‐1 (clone 1B1, hybridoma supernatant, 1:500) 23, polyclonal rabbit anti‐human GBP‐1 1:5000 23, polyclonal rabbit anti‐human caspase‐1 p20 1:200 (sc‐622; Santa Cruz Biotechnology, Dallas, TX, USA), monoclonal rabbit anti‐human caspase‐3 1:1000 (8G10, Cell Signaling, Danvers, MA, USA), monoclonal mouse anti‐human caspase‐5 1:1000 (4F7, MBL, Woburn, MA, USA), monoclonal mouse anti‐human IL‐1β 1:1000 (MAB201; R&D Systems, Minneapolis, MN, USA), monoclonal mouse anti‐Flag 1:2500 (M2; Sigma‐Aldrich), polyclonal rabbit anti‐Flag 1:1000 (F7425; Sigma‐Aldrich) and monoclonal mouse anti‐human GAPDH 1:40,000 (6C5; Millipore, Schwalbach, Germany). All horseradish peroxidase‐conjugated secondary antibodies were diluted 1:5000 and purchased from GE Healthcare. Protein detection was performed using the enhanced chemiluminescence Western blot detection system (ECL; GE Healthcare, Little Chalfont, UK) and Rx‐films (Fuji, Tokyo, Japan) or a chemoluminescence detector (Amersham Imager 600, GE Healthcare). Quantification of Western blot band intensity was performed using the ImageJ software 40.

### Lactate dehydrogenase activity assay

Cellular toxicity was analysed by determination of lactate dehydrogenase (LDH) activity in the cell supernatants using the CytoTox 96 non‐radioactive cytotoxicity assay (Promega, Mannheim, Germany). The assay was performed according to the manufacturer's protocol.

### GBP‐1‐ELISA

MaxiSorp immunoplates (Nunc) were coated overnight with 1 μg/ml of purified rat anti‐GBP‐1 monoclonal antibody (clone 1B1 1, 30). Plates were rinsed with PBS–0.1% Tween‐20 (PBS‐T), blocked with PBS–1% skim milk for 30 min and incubated with the samples in triplicates for 2 hrs. Cell culture supernatants were used undiluted, and samples of human CSF were diluted 1:8 in PBS. Subsequently, the plates were incubated with rat anti‐GBP‐1 IgG2a monoclonal antibody for 2 hrs (clone 6E6, 1:11, detection antibody, own laboratory), with a biotinylated anti‐rat IgG2a antibody for 1 hr (clone TIB173, 1:500 41) and with avidin‐horseradish peroxidase (1:2000) for 1 hr (Vector laboratories, Peterborough, UK). Next, 1‐Step Ultra TMB‐ELISA Substrate (Thermo Scientific, Whaltham, MA, USA) was added for 30 min., the colour reaction was stopped by addition of 2 M sulphuric acid and quantified at 450 nm in a microplate reader (model 680; Bio‐Rad, Munich, Germany). Standard curves were obtained with recombinant purified GBP‐1‐His protein (0–100 ng/ml), either diluted in EBM‐2 (cell culture supernatant samples) or in PBS (liquor samples). The standard curves of the ELISA were linear up to 100 ng/ml of GBP‐1.

### Caspase cleavage assay

To assess *in vitro* caspase cleavage, 500 ng of purified recombinant human GBP‐1 was incubated for 3 hrs in cleavage buffer (50 mM HEPES pH 7.2, 50 mM NaCl, 0.1% CHAPS, 10 mM EDTA, 5% glycerol and 10 mM DTT) with different amounts of recombinant human caspase‐1, caspase‐3, caspase‐4 or caspase‐5 (BioVision, Mountain View, CA, USA) with or without 0.5–1 mM Z‐VAD‐fmk or Z‐YVAD‐fmk. The reaction products were analysed by Western blot.

### Mass spectrometric analysis of proteins

Proteins were isolated from SyproRuby (Invitrogen, Karlsruhe, Germany)‐stained SDS‐PAGE gels and subjected to commercial MS‐MALDI analysis (Toplab GmbH, Martinsried, Germany).

### *In silico* analyses

The cleavage site prediction was performed with ‘PeptideCutter’ on the exPASy server (http://www.expasy.org/tools/peptidecutter/) and the Grabcas application 42. The alignment of GBP‐1 (accession number: NP_002044) with different GBPs (GBP‐2: NP_004111; GBP‐3: NP_060754; GBP‐4: NP_443173; GBP‐5: AAL02055; GBP‐6: NP_940862; GBP‐7: Q8N8V2; GBP of *Pongo pygmaeus*: Q5RBE1; GBP of *Cercopithecus aethiops*: Q5D1D6; GBP of *Bos taurus*: Q0II27) was performed with Vector NTI (Invitrogen).

### Clinical samples

Lumbar punctures were performed for diagnostic purposes after informed consent of patients and in agreement with the recommendations of the local ethics committee of the University of Erlangen‐Nuremberg. After centrifugation, the CSF samples were stored at −80°C until analysis. In total, 41 patients were analysed: 20 of them were diagnosed to suffer from bacterial meningitis and 21 were negative controls (non‐infectious diseases). The patients with bacterial meningitis were infected with *Streptococcus equi* (*n* = 1), *Streptococcus pneumoniae* (*n* = 5), *Neisseria meningitidis* (*n* = 2), *Escherichia coli* (*n* = 1), *Listeria monocytogenes* (*n* = 2), *Acinetobacter Iwoffii* (*n* = 1), *Staphylococcus epidermidis* (*n* = 3), *Staphylococcus aureus* (*n* = 2), *Enterococcus faecium* (*n* = 1) and *Haemophilus influenzae* (*n* = 2). Sex distribution and age were not statistically different between patients with bacterial meningitis (mean age 51.9 years, range 13–78; female/male ratio 0.82) and controls (mean age 44.6 years, range 0.16–76; female/male ratio 0.91). In the meningitis *versus* control patients, the mean leucocyte counts (per μl CSF) were 1914.1 (range 0–11,105; values in four patients missing or not determined) *versus* 270 (range 0–2871; 2 values missing), the mean erythrocyte counts (per μl CSF) were 323.5 (range 0–2100; 6 values missing) *versus* 1542.7 (range 0–11,000; 3 values missing) and the mean protein amounts (mg/ml CSF) were 1768.2 (range 132–6229; 7 values missing) *versus* 775.6 (range 151–1961; 4 values missing).

First, 50 μl of the samples was subjected to the GBP‐1‐ELISA to determine the total GBP‐1 levels. Second, up to 400 μl of CSF was subjected to whole‐protein acetone precipitation followed by anti‐GBP‐1 Western blot.

## Results

### A 47‐kD cleavage product, corresponding to the C‐terminal helical part of GBP‐1, is present in the cell culture supernatants of IFN‐γ‐treated endothelial cells

**Figure 1:**
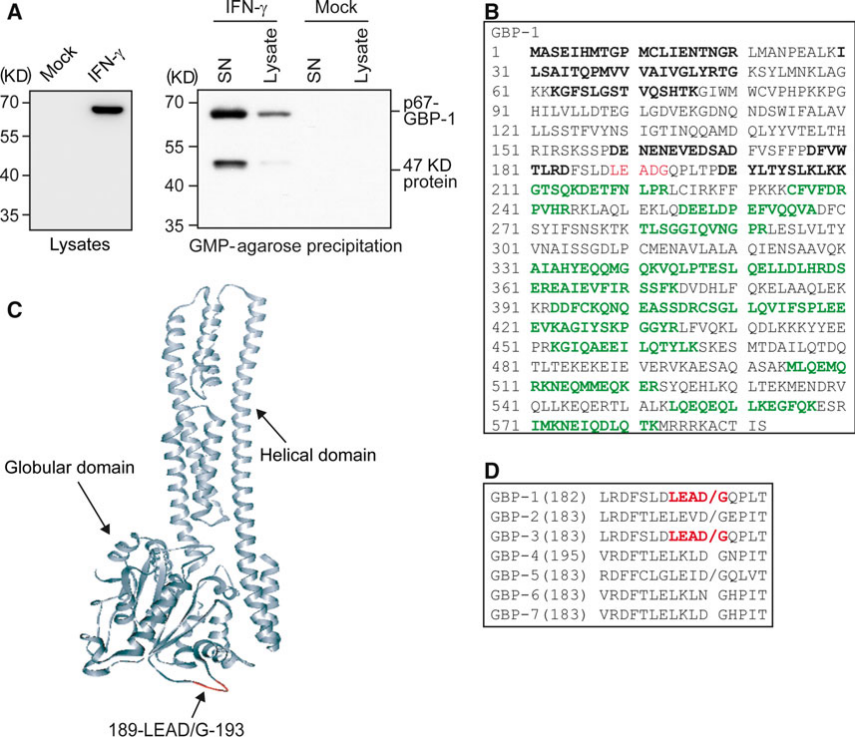
A 47‐kD protein fragment corresponding to the C‐terminal part of GBP‐1 is detected in precipitated cell culture supernatants of IFN‐γ‐treated HUVEC. (**A**) HUVECs were either untreated (mock) or stimulated for 24 hrs with 100 U/ml IFN‐γ. Cell lysates were analysed by Western blot (left panel) using a monoclonal anti‐human GBP‐1 antibody. In addition, cell lysates and supernatants were subjected to GMP‐agarose precipitation under identical conditions. The precipitates were analysed by Western blot using a monoclonal anti‐human GBP‐1 antibody (right panel). Two specific bands were detected in the supernatant. (**B**) The 67‐kD and 47‐kD bands observed in Figure 1B were cleaved in‐gel by trypsin and analysed by MALDI‐TOF. The measured peptide masses were used for a search with ProFound against the NCBI database. Identified peptides from the p67‐GBP‐1 band are depicted in bold. Peptides derived from the 47‐kD band are depicted in bold and green. The caspase‐1 and caspase‐5 cleavage sites are labelled in red. (**C**) The position of 189‐LEAD/G‐193 is highlighted (red) in the crystal structure of GBP‐1 19. (**D**) Protein sequence alignment of human GBPs was performed to investigate the presence of the caspase cleavage site 189‐LEAD/G‐193 (bold, red). Numbers of the amino acids at the beginning of the corresponding areas are given in brackets. A potential cleavage site of either caspase‐1, caspase‐5 or both is indicated by a slash.

In order to investigate whether secreted GBP‐1 binds guanylate, the supernatant of IFN‐γ‐stimulated (100 U/ml, 24 hrs) HUVEC was subjected to GMP‐agarose precipitation. IFN‐γ treatment induced GBP‐1 expression, and full‐length GBP‐1 was precipitated from the supernatant of IFN‐γ‐stimulated cells, showing that the protein had retained its guanylate‐binding ability. No GBP‐1‐specific signal was obtained from the supernatant and cell lysate of unstimulated control cells (Fig. 1A; mock). In addition to the 67‐kD full‐length GBP‐1 protein (p67‐GBP‐1), a 47‐kD protein fragment was detected in the cell culture supernatant of IFN‐γ‐treated cells by Western blot analyses using a monoclonal anti‐human GBP‐1 antibody (clone 1B1 1) (Fig. 1A, IFN‐γ, SN). However, the 47‐kD protein was not detectable in the cell lysates (Fig. 1A; left panel, lysates), and only a faint band was visible when lysates were precipitated with GMP‐agarose (Fig. 1A, right panel, IFN‐γ, lysate), indicating that the 47‐kD fragment is primarily secreted but may arise from an intracellular cleavage. The epitope of the monoclonal anti‐human GBP‐1 antibody 1B1 is located within the C‐terminal helical domain of GBP‐1 (Fig. 1C and Fig. S1). This suggested that the 47‐kD protein might be a C‐terminal cleavage fragment of GBP‐1. In order to determine unequivocally the identity of the secreted 67‐kD and 47‐kD proteins, GMP‐agarose precipitates were subjected to an in‐gel digestion with trypsin and were analysed by MALDI‐TOF. The identified peptides from the 67‐kD band were randomly distributed within the whole GBP‐1 sequence (sequence coverage: 33%) (Fig. 1B, bold). Peptides derived from the 47‐kD band covered the amino acids 211‐582 of the full‐length GBP‐1 sequence (Fig. 1B, bold and green). This confirmed that the 47‐kD protein is a C‐terminal product of full‐length GBP‐1. In the following, this 47‐kD protein fragment will be termed p47‐GBP‐1.

### Inflammatory caspase cleavage activity is required for p47‐GBP‐1 generation and secretion

Computer‐assisted analysis of cleavage sites for proteases identified a unique motif, which is potentially targeted by the inflammatory caspases‐1, ‐4 and ‐5 43 at the position 189‐LEAD/G‐193 (the slash indicates the caspase cleavage site) in GBP‐1 (Fig. 1C and D, red). This putative cleavage motif is localized at the C‐terminal side of the third GTPase binding motif and is exposed on the surface of the protein (Fig. 1C, red, arrow). Sequence comparison revealed that in human GBPs, the LEAD/G cleavage site is only present in GBP‐1 and its closest homologue GBP‐3, but in none of the other human GBPs (Fig. 1D, red). An identical cleavage site was detected in the GBPs of *Pongo pygmaeus* (orangutan), *Cercopithecus aethiops* (green monkey) and *Bos taurus* (cow) (data not shown).

**Figure 2:**
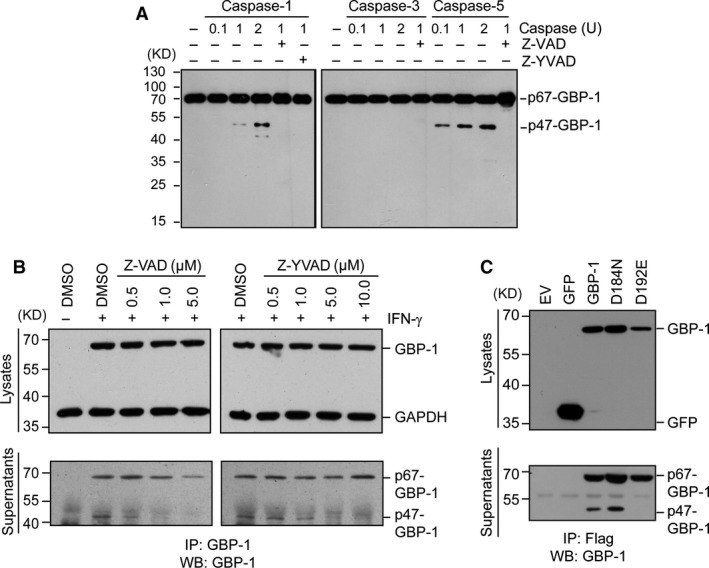
Cleavage and release of p47‐GBP‐1 in the cell culture supernatant of HUVECs depend on inflammatory caspase activity. (**A**) Recombinant GBP‐1 (500 ng) purified from *E. coli* was incubated without (control) or with recombinant caspase‐1, caspase‐3 or caspase‐5 for 3 hrs at 37°C at the indicated concentrations in the absence or presence of the pan‐caspase inhibitor Z‐VAD‐fmk (Z‐VAD, 1 mM) and the caspase‐1 inhibitor Z‐YVAD‐fmk (Z‐YVAD, 1 mM). The reaction products were separated on a SDS‐PAGE and analysed by Western blot using a polyclonal anti‐human GBP‐1 antibody. (**B**) HUVECs were treated with IFN‐γ (100 U/ml) in the presence or absence of Z‐VAD‐fmk or Z‐YVAD‐fmk (Z‐YVAD) as indicated. Lysates were harvested 48 hrs after treatment and subjected to Western blot analyses with a monoclonal anti‐GBP‐1 antibody (lysates, GBP‐1) and an anti‐GAPDH antibody (lysates, GAPDH) as a loading control. Cell culture supernatants were subjected to immunoprecipitation using a polyclonal anti‐GBP‐1 antibody. The precipitated proteins were analysed by immunoblotting using a monoclonal anti‐GBP‐1 antibody. (**C**) HUVECs were transiently transfected with different expression plasmids encoding Flag‐tagged wild‐type GBP‐1, D184N‐GBP‐1 (mutant with diminished GTPase activity), D192E‐GBP‐1 (mutant with inactivated caspase‐1/‐5 cleavage motif) and GFP (negative control). Intracellular expression of the different proteins was analysed by Western blot of the cell lysates using a monoclonal anti‐Flag antibody. Cell culture supernatants were subjected to an anti‐Flag immunoprecipitation and subsequent Western blot analysis using a monoclonal anti‐human GBP‐1 antibody.

Therefore, it was investigated whether p47‐GBP‐1 is generated from full‐length recombinant GBP‐1 in the presence of caspases using an *in vitro* cleavage assay (Fig. 2A). Incubation of recombinant GBP‐1 with inflammatory caspases‐1 and ‐5 generated a cleavage product of 47‐kD, whereas no cleavage was observed with the executer caspase‐3, known for its role in the extrinsic and intrinsic apoptosis pathways (Fig. 2A). Furthermore, the 47‐kD protein fragment was not detected in the presence of the pan‐caspase inhibitor Z‐VAD‐fmk, of the caspase‐1‐specific inhibitor Z‐YVAD‐fmk or when caspases were omitted (Fig. 2A). Cleavage of GBP‐1 by caspase‐4 resulted in the formation of a 40‐kD fragment (Fig. S2). These data indicated that the inflammatory caspases‐1 and ‐5 are responsible for the formation of p47‐GBP‐1. To confirm that p47‐GBP‐1 is generated by caspase‐1/‐5 cleavage in the cellular context, HUVECs were stimulated with IFN‐γ in the presence or absence of the pan‐caspases inhibitor Z‐VAD‐fmk or of the caspase‐1‐specific inhibitor Z‐YVAD‐fmk (Fig. 2B), and the cell culture supernatants were precipitated with a polyclonal anti‐GBP‐1 antibody (Fig. 2B). Treatment with both, Z‐VAD‐fmk and Z‐YVAD‐fmk, reduced the amount of extracellular p47‐GBP‐1 in a concentration‐dependent manner (Fig. 2B; supernatants, p47‐GBP‐1 and Fig. S2B), but did not affect the intracellular expression of GBP‐1 (Fig. 2B; lysates, GBP‐1). Comparable levels of GAPDH showed that similar amounts of proteins were blotted onto the membrane (Fig 2B; lysates, GAPDH). Of note, the secretion of full‐length p67‐GBP‐1 was also reduced at the highest concentration of Z‐VAD‐fmk (Fig. 2B; left panel, supernatants, p67‐GBP‐1). However, normalization of the p47‐GBP‐1 amount to precipitated p67‐GBP‐1 revealed that the secretion of p47‐GBP‐1 was more strongly decreased than the secretion of p67‐GBP‐1 by treatment with the pan‐caspase inhibitor (Fig. S2B). These results indicated that caspase activity is necessary for the generation of p47‐GBP‐1.

As multiple attempts to specifically down‐regulate caspase‐1 and caspase‐5 in primary endothelial cells by transient or stable RNA silencing have remained unsuccessful (data not shown), we generated a GBP‐1 mutant with an inactivated caspase cleavage motif in order to further investigate whether p47‐GBP‐1 is produced through cleavage by inflammatory caspases. For this purpose, the aspartic acid 192 was changed into glutamic acid (D192E) and the resulting mutant protein was expressed with an N‐terminal Flag tag in HUVEC (Fig. 2C, D192E). Cells expressing Flag‐tagged wild‐type GBP‐1 or GTPase‐deficient GBP‐1 D184N (Fig. 2C, GBP‐1 and D184N) were used as control. After transient transfection, p47‐GBP‐1 was clearly detected in the supernatant of control cells (Fig. 2C, GBP‐1 and D184N). However, no cleaved form could be detected when the D192E mutant was expressed (Fig. 2C, supernatants, D192E). These results demonstrated that cleavage of GBP‐1 at the caspase recognition site 189‐LEAD/G‐193 is required for the generation and secretion of p47‐GBP‐1.

### P47‐GBP‐1 is the most abundant form of secreted GBP‐1 *in vitro*

The 47‐kD fragment of the C‐terminal part of GBP‐1 does not contain the GMP‐binding motif, which resides in the N‐terminal part of the full‐length protein. Using a yeast two‐hybrid approach, it was previously shown that full‐length GBP‐1 is able to interact with its own C‐terminal helical part 44. This suggested that the 47‐kD cleavage fragment was co‐purified during GMP precipitation by binding to the full‐length protein. Similarly, p47‐GBP‐1 was not directly targeted by Flag immunoprecipitation of Flag‐tagged GBP‐1 (Fig. 2C) indicating that it was co‐precipitated also in this case.

**Figure 3:**
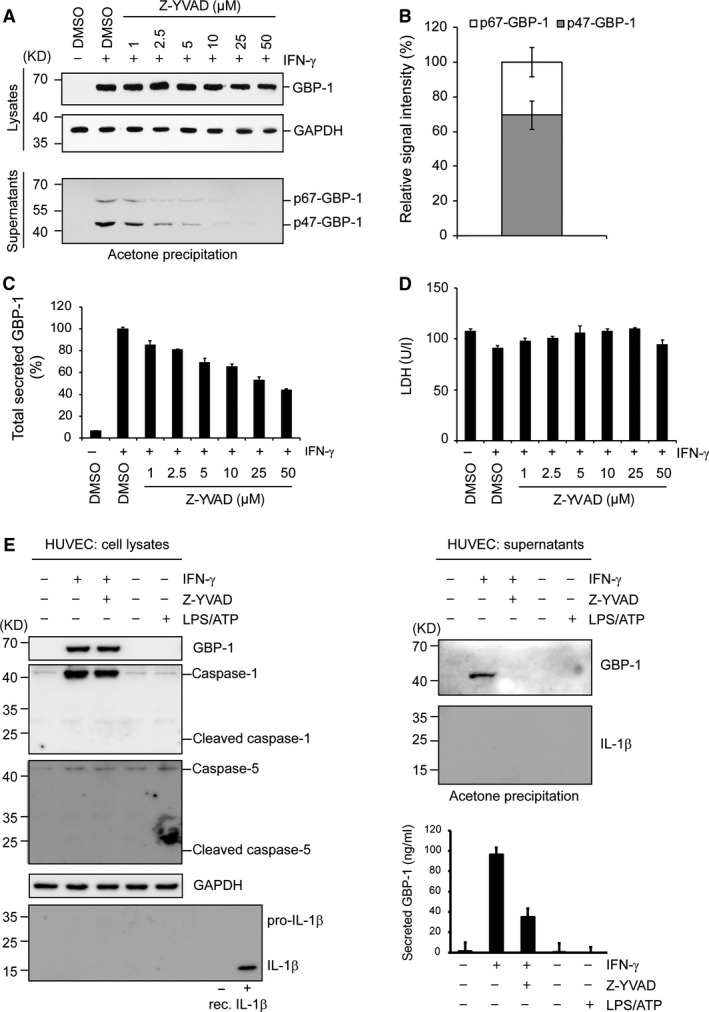
p47‐GBP‐1 is the most abundantly secreted form of GBP‐1 *in vitro* and is generated in an inflammasome‐independent manner. (**A**) HUVECs were untreated or treated with IFN‐γ (100 U/ml) for 6 hrs before the caspase‐1 inhibitor Z‐YVAD‐fmk (Z‐YVAD) was added for 18 hrs at the indicated concentrations. DMSO, the solvent of Z‐YVAD‐fmk, was used as negative control. Upper panel: Lysates were harvested and subjected to Western blot analyses with a monoclonal anti‐GBP‐1 antibody (lysates, GBP‐1) and an anti‐GAPDH antibody (lysates, GAPDH) as a loading control. Lower panel: Cell culture supernatants were subjected to acetone precipitation. The precipitated proteins were analysed by immunoblotting using a monoclonal anti‐GBP‐1 antibody (supernatants, p67‐GBP‐1 and p47‐GBP‐1). (**B**) The relative amount of p47‐GBP‐1 and p67‐GBP‐1 secreted by HUVECs after treatment with IFN‐γ (100 U/ml) was quantified for three different Western blots from acetone‐precipitated supernatants using the ImageJ software, and the results are presented in percent of total secreted GBP‐1. (**C**) The total concentration of secreted GBP‐1 was quantified by ELISA in the cell culture supernatants. The amount of GBP‐1 secreted after treatment with IFN‐γ and in the absence of inhibitor (233 ng/ml) was set to 100%. (**D**) Non‐specific release of proteins due to cell death was determined by measurement of the activity of the intracellular enzyme lactate dehydrogenase (LDH) in the supernatants. (**E**) HUVECs were untreated or treated with IFN‐γ (100 U/ml) or LPS (1 μg/ml) for 6 hrs before the caspase‐1 inhibitor Z‐YVAD‐fmk (Z‐YVAD, 50 μM) was added and further incubated for 30 hrs and 16 hrs, respectively. DMSO, the solvent of Z‐YVAD‐fmk, was used as negative control. ATP (5 mM) was added 30 min. before harvesting of cell lysates and supernatants. Left panel: Lysates were harvested and subjected to Western blot analysis. Recombinant IL‐1β (rec. IL‐1β, 2 ng) was used as a detection control, and GAPDH was used as loading control. Right panel: Cell culture supernatants were subjected to acetone precipitation followed by Western blot and the total concentration of secreted GBP‐1 (ng/ml) was quantified by ELISA.

The detection of only those p47‐GBP‐1 molecules which were associated and co‐precipitated with p67‐GBP‐1 may lead to an underestimation of the amount of extracellular p47‐GBP‐1. To determine the quantitative relation between secreted p67‐ and p47‐GBP‐1, IFN‐γ‐treated HUVECs culture supernatants were precipitated as a whole using acetone. This approach showed that p47‐GBP‐1 is present extracellularly in the cell supernatants in higher amounts than p67‐GBP‐1 (Fig. 3A and B). Treatment with increasing concentrations of Z‐YVAD‐fmk resulted in the inhibition of both p47‐ and p67‐GBP‐1 secretion by endothelial cells (Fig. 3A), while the intracellular amounts of GBP‐1 remained constant (as shown by normalization to the GAPDH levels). The inhibition of GBP‐1 secretion by Z‐YVAD‐fmk was confirmed by quantification of the amount of secreted GBP‐1 using a specific GBP‐1‐ELISA based on the 1B1 monoclonal antibody, which detects both p67‐ and p47‐GBP‐1 (Fig. 3C). Of note, the release of the intracellular enzyme LDH was not influenced by treatment with Z‐YVAD‐fmk, indicating that caspase‐1 inhibition did not affect cellular toxicity and that p47‐GBP‐1 is not passively released as a product of cell death (Fig. 3D).

### P47‐GBP‐1 is generated in an inflammasome‐independent manner

Inflammatory caspases are known to be proteolytically activated within large multi‐protein complexes termed inflammasomes 35. Inflammasome activation has been shown to occur primarily in myeloid cells, notably in monocytes/macrophages, upon engagement of pattern recognition receptors 45. Inflammasome activation results in the processing of caspase‐1, which thereby becomes active and able to cleave pro‐IL‐1β or pro‐IL‐18 36. Cleaved IL‐1β and IL‐18 are then secreted *via* a non‐classical pathway 45. In monocytes, efficient activation of the inflammasome can be triggered by ATP after priming of the cells with lipopolysaccharide 35, 46, 47.

To address whether the inflammasome is activated by IFN‐γ in HUVECs, we investigated caspase‐1 and caspase‐5 expression and cleavage. Expression of caspase‐1 was highly induced by IFN‐γ whereas caspase‐5 was constitutively expressed at a low level in these cells (Fig. 3E). No proteolytic cleavage of both caspases could be detected after treatment with either IFN‐[gamma] or LPS+ATP (Fig. 3E). In contrast, cleavage‐associated activation of caspase‐1 could be detected in THP‐1 cells (Fig. S3). Detection of processing and secretion of pro‐IL‐1β was attempted as an additional positive control of inflammasome activation, but this cytokine was not expressed in HUVECs (Fig. 3E). On the contrary, expression and secretion of IL1‐[beta] was induced by treatment with LPS+ATP in THP‐1 cells (Fig. S3). Highly increased expression of caspase‐1 in IFN‐γ‐treated HUVECs suggested that caspase‐1 rather than caspase‐5 might be responsible for GBP‐1 cleavage in endothelial cells. Taken together, these data confirmed that secretion of GBP‐1 is cell‐type specific and indicate that it occurs in a caspase‐1‐dependent manner but independently of inflammasome activation.

### P47‐GBP‐1 is released through a non‐classical caspase‐1‐dependent secretion pathway

**Figure 4:**
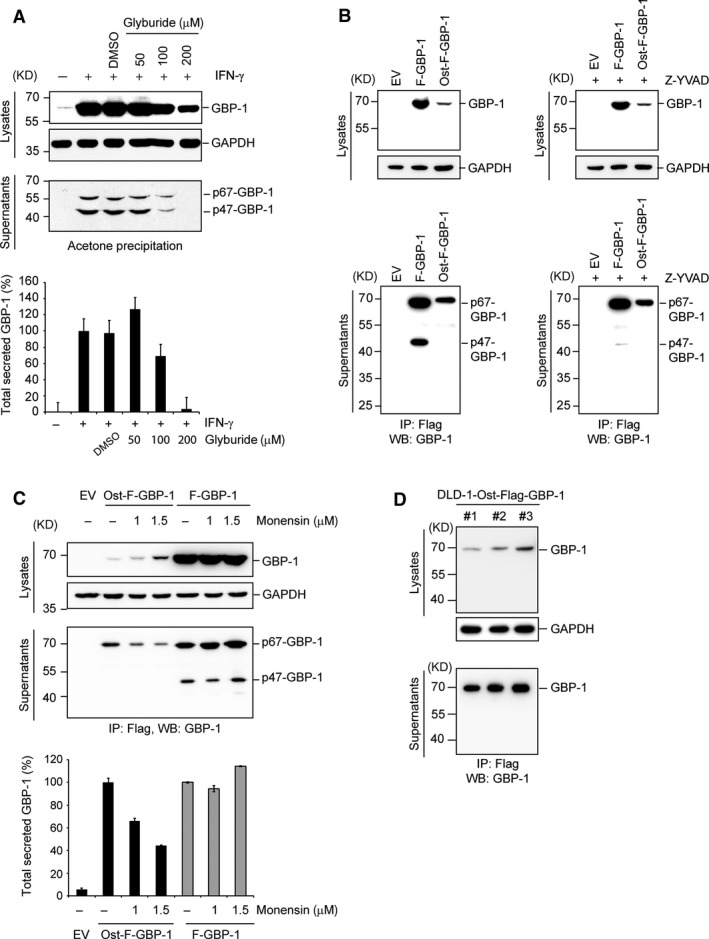
p47‐GBP‐1 is secreted through a non‐conventional caspase‐1‐dependent secretion pathway. (**A**) HUVECs were treated with increasing concentrations of the non‐classical secretion inhibitor glyburide for 2 hrs before IFN‐γ (100 U/ml) was added for 46 hrs. As negative control DMSO, the respective solvent of glyburide was used. Lysates were harvested and subjected to Western blot analyses with a monoclonal anti‐GBP‐1 antibody (Lysates, GBP‐1) and an anti‐GAPDH antibody (Lysates, GAPDH) as a loading control. Cell culture supernatants were subjected to acetone precipitation and analysed by immunoblotting using a monoclonal anti‐GBP‐1 antibody (Supernatants, p67‐GBP‐1 and p47‐GBP‐1). The total concentration of GBP‐1 in the cell culture supernatants was assessed by ELISA (lower panel). (**B**) HUVEC were transiently transfected with expression plasmids encoding Flag‐tagged wild‐type GBP‐1 or a mutant containing the classical secretion signal peptide of osteonectin (Ost) in the absence (left panels) or presence (right panels) of Z‐YVAD (50 μM). The corresponding empty vector (EV) was used as control. Intracellular expression of the different proteins was analysed by Western blot of the cell lysates using a monoclonal anti‐Flag antibody. Cell culture supernatants were subjected to an anti‐Flag immunoprecipitation and subsequent Western blot analysis using a monoclonal anti‐human GBP‐1 antibody. (**C**) HUVECs were transiently transfected to express Ost‐F‐GBP‐1 or F‐GBP‐1. The empty vector (EV) was used as negative control. Cells were treated with increasing concentrations of monensin, an inhibitor of the classical secretion pathway, or with methanol, the corresponding solvent (‐). Lysates were harvested and subjected to Western blot analyses with a monoclonal anti‐Flag antibody (Lysates, GBP‐1) and an anti‐GAPDH antibody (Lysates, GAPDH) as a loading control. Cell culture supernatants were subjected to anti‐Flag precipitation and analysed by immunoblotting using a monoclonal anti‐GBP‐1 antibody (Supernatants, p67‐GBP‐1 and p47‐GBP‐1). The total concentration of GBP‐1 in the cell culture supernatants was assessed by ELISA (lower panel) and is expressed in percent of secreted GBP‐1 in Ost‐F‐GBP‐1‐ (black) or F‐GBP‐1 (grey)‐untreated cells. (**D**) DLD‐1 cells were stably transfected with expression plasmid encoding the Ost‐Flag‐GBP‐1 mutant. Three independent stable clones were investigated (#1, #2 and #3). Cell culture supernatants were subjected to an anti‐Flag immunoprecipitation. Western blot analysis of intra‐ and extracellular expression was performed using a monoclonal anti‐human GBP‐1 antibody. GAPDH was used as a loading control.

GBP‐1 does not contain a leader sequence for classical secretion. Using whole GBP‐1 ELISA, we previously showed that the secretion of GBP‐1 is impaired by treatment with glyburide, an inhibitor of non‐classical secretion 30. Next, we investigated whether p47‐GBP‐1 is also released *via* a non‐classical secretion pathway. Treatment of IFN‐γ‐stimulated HUVEC with glyburide inhibited the secretion of p67‐ and p47‐GBP‐1 in a dose‐dependent manner as observed by Western blot of acetone‐precipitated supernatants and ELISA (Fig. 4A), showing that p47‐GBP‐1, too, is secreted through a non‐classical pathway. Of note, at the highest glyburide concentration, the intracellular amount of GBP‐1 dropped to 41% as compared to the control in the absence of glyburide (IFN‐γ and DMSO). In contrast, the extracellular amount of GBP‐1 decreased at the same experimental point down to 3.6% (ELISA) and 5.9% (acetone precipitation) as compared to the control indicating a more prominent impact of glyburide on GBP‐1 secretion as compared to its impact on intracellular GBP‐1 expression. In addition, we constructed vectors for transient and stable expression of Flag‐GBP‐1 fused with the leader peptide of Osteonectin in N‐terminal (Ost‐F‐GBP‐1) in order to reroute the secretion into the classical pathway. After ectopic expression of F‐GBP‐1 and Ost‐F‐GBP‐1 in HUVECs, the p47 fragment could only be detected in the cell supernatant of HUVECs expressing F‐GBP‐1 (Fig. 4B, left panel, IP:Flag), where its secretion was inhibited by treatment with the caspase‐1 inhibitor Z‐YVAD‐fmk (Fig. 4B, right panel, IP:Flag). The addition of monensin, an inhibitor of the classical secretion pathway, reduced the secretion of Ost‐F‐GBP‐1, but did not affect the release of F‐GBP‐1 nor its cleavage (Fig. 4C). Finally, a colorectal cancer cell line (DLD‐1) stably transfected with the Ost‐F‐GBP‐1 expression plasmid was able to express and secrete high amounts of the full‐length protein but not p47‐GBP‐1 (Fig. 4D), indicating an interdependence between protein cleavage and secretion pathway. These results show that the generation and secretion of p47‐GBP‐1 depends on unconventional secretion mechanisms involving caspase‐1 activity. Moreover, this indicates that the cleavage of GBP‐1 does not ensue from unspecific proteolysis in the cell culture supernatant as it was observed in the supernatant of F‐GBP‐1 but not Ost‐F‐GBP‐1‐expressing cells (Fig. 4B and C).

### *In vivo*, p47‐GBP‐1 is detected in the CSF of bacterial meningitis patients

**Figure 5:**
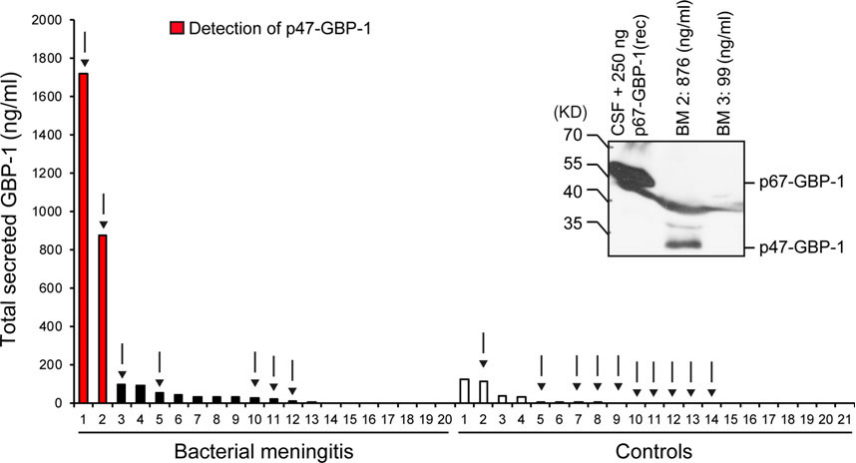
*In vivo,* p47‐GBP‐1 is detected in the CSF of patients with bacterial meningitis. Left panel: total GBP‐1 levels were determined by ELISA in liquor samples of patients with bacterial meningitis (*n* = 20, filled bars) and control patients with non‐infectious diseases (*n* = 21, empty bars). Samples with enough material remaining after ELISA measurement were subjected to whole‐protein precipitation and WB (arrows). CSF samples, in which p47‐GBP‐1 was detected after precipitation and WB, are marked in red. Right panel: detection of p47‐GBP‐1 in two patients with bacterial meningitis compared to a GBP‐1‐negative CSF sample spiked with 250 ng of recombinant p67‐GBP‐1 as a control after whole‐protein acetone precipitation followed by WB and detection with anti‐GBP‐1 monoclonal antibody.

Finally, it was analysed whether the p47‐GBP‐1 protein fragment is detectable *in vivo*. We previously showed that GBP‐1 can be detected by ELISA in the CSF of patients with bacterial meningitis 30, 31. To investigate which form of the protein was present *in vivo*, the CSF of patients with bacterial meningitis was analysed using GBP‐1‐ELISA followed by whole‐protein acetone precipitation. For this purpose, the total GBP‐1 concentration was determined by GBP‐1‐ELISA in CSF samples of patients with bacterial meningitis (*n* = 20) and patients who were suffering from other non‐infectious diseases (*n* = 21) and was found to be increased in the meningitis patients compared to the controls (Fig. 5, diagram). Next, CSF probes were subjected to whole‐protein acetone precipitation. The precipitation was initially standardized using CSF samples negative for GBP‐1, as detected by ELISA, that were supplemented with recombinant GBP‐1. During the analysis of these samples, we noted that the running behaviour of p67‐GBP‐1 in the gel was altered towards lower size appearance most likely due the presence of high protein amounts in the CSF samples as compared to cell culture extracts (Fig. 5, right panel, p67‐GBP‐1 recombinant). The p47‐GBP‐1 protein fragment was detected after acetone precipitation in the samples of the meningitis patients with the highest amounts of total GBP‐1 determined by ELISA (Fig. 5, red bars and right panel). On the contrary, the p67‐GBP‐1 form was not or only faintly visible (Fig. 4, right panel). The pathogenic agent involved in those cases was *Streptococcus pneumoniae* and *Staphylococcus aureus*, respectively. There was no association between the infectious agent and the presence of p47‐GBP‐1 in the CSF, suggesting that the latter rather depends on the concentration of the protein and on the detection limit. Overall, these results indicated that p47‐GBP‐1 is the predominant form of secreted GBP‐1 also *in vivo*.

## Discussion

It has been shown previously that GBP‐1 is secreted by endothelial cells but not by any other cell type tested including fibroblasts, smooth muscle cells or tumor cell lines 30. Here, we identified an additional form of secreted GBP‐1 corresponding to a C‐terminal cleavage fragment of the protein (aa 193‐592) in the supernatants besides the full‐length p67‐GBP‐1. As this 47‐kD fragment lacks the N‐terminal part of the globular domain containing the guanylate binding function, it seemed likely that it was indirectly co‐precipitated *via* its interaction with full‐length GBP‐1 during GMP‐agarose precipitation. Indeed, GBP‐1 has the property to form oligomers, and oligomerization activates the GTPase activity of the protein 48. More precisely, it has been shown that GBP‐1 forms homodimers through its N‐terminal globular domain and tetramers *via* the C‐terminal helical domain 44, 48. The helical domain of GBP‐1, which roughly corresponds to the p47 fragment described here, has the ability to bind the full‐length protein, confirming our data 44. We were able to detect both the p47 and p67 forms of GBP‐1 in the cell culture supernatants of endothelial cells by GMP or immunoprecipitation against p67‐GBP‐1. However, using whole‐protein acetone precipitation, we found that p47‐GBP‐1 represented the most prominent form (roughly 70%) of GBP‐1 secreted by endothelial cells. P47‐GBP‐1 was also detected as the predominant form in CSF samples of some patients with bacterial meningitis, whereas p67‐GBP‐1 was not or only faintly visible. Altogether, these data suggest that p47‐GBP‐1 is generated both *in vitro* and *in vivo*, and might therefore have a biological relevant role. Actually, intracellular expression of the helical domain of GBP‐1 (aa 289‐592), which is also present in p47‐GBP‐1 (aa 193‐592) and lacks GTPase activity, has previously been shown to inhibit the proliferation of endothelial cells 23. We performed preliminary analyses to determine whether secreted p47‐GBP‐1 might contribute to the inhibitory effects of GBP‐1 on cell proliferation. Using conditioned medium (CM), we observed that the proliferation of HUVEC was reduced in the presence of p47‐GBP‐1 (data not shown). This suggested that p47‐GBP‐1 may act as a paracrine inhibitor on endothelial cell proliferation.

Several different experimental approaches showed that p47‐GBP‐1 originates from the full‐length protein following cleavage by inflammatory caspases: (*i*) The incubation of recombinant GBP‐1 with caspase‐1 and caspase‐5, but not caspase‐4, generated p47‐GBP‐1; (*ii*) treatment of GBP‐1‐expressing endothelial cells with caspase inhibitors abrogated the release of p47‐GBP‐1; and (*iii*) the mutation of the potential caspase‐1/‐5 cleavage site blocked the formation of p47‐GBP‐1. Cleavage by caspase‐1/‐5 was responsible for the formation of p47‐GBP‐1, and the inhibition of caspase‐1 activity alone by Z‐YVAD‐fmk was sufficient to block the secretion of both p47‐ and p67‐GBP‐1 from endothelial cells. These findings indicated that caspase‐1 activity is necessary for both the cleavage and the secretion of GBP‐1. However, the question still remained whether the cleavage of GBP‐1 only occurs in the cell culture supernatant and p47‐GBP‐1 represents a mere by‐product of GBP‐1 secretion. In fact, Tschopp and coworkers showed that caspase‐1 is activated in response to inflammatory signals at potassium concentrations below 90 mM 49. Thus, GBP‐1 could be cleaved by caspase‐1 in the cell culture supernatant, where both proteins are present 30, 50, 51, and where the potassium concentration is very low (4 mM). To address this issue, we investigated whether the presence in the cell culture supernatant of GBP‐1 is sufficient for the generation of the p47 form. To this purpose, the secretion of GBP‐1 was forced *via* the classical secretion pathway by the addition of a leader peptide. After expression of a fusion protein containing the signal peptide of osteonectin upstream to the Flag‐tagged full‐length GBP‐1 sequence, high amounts of p67‐GBP‐1 were present in the cell culture supernatant but no p47‐GBP‐1 could be detected, both in transiently transfected endothelial cells and in stably transfected DLD‐1 cells. This confirmed that the generation of p47‐GBP‐1 is not occurring spontaneously in the cell culture supernatant. Furthermore, the amount of extracellular p67‐GBP‐1 was not increased when cleavage was inhibited, either chemically or by expression of a mutant GBP‐1, indicating that p47‐GBP‐1 is not produced by cleavage of already secreted p67‐GBP‐1, and suggesting that cleavage and secretion of p47‐GBP‐1 are inextricable. The fact that p47‐GBP‐1 is only observed in cell lysates after precipitation suggests that the cleavage products are rapidly secreted and processing of GBP‐1 occurs at the plasma membrane in the course of the secretion process, similarly to what has been described for IL‐1β 46.

An additional concern was that the generation of p47‐GBP‐1 might constitute a by‐product of cell death. The fact that GBP‐1 is not a target of caspase‐3, the apoptosis effector caspase, strongly argues against this possibility. In addition, neither IFN‐γ, which induced the formation of p47‐GBP‐1, nor a caspase‐1 inhibitor, which inhibited the generation of p47‐GBP‐1, altered the viability of HUVECs. Furthermore, similar cytotoxicity levels were observed in endothelial cells transfected with an empty vector and in cells expressing Flag‐GBP‐1 or Ost‐Flag‐GBP‐1, while p47‐GBP‐1 was only observed in the supernatant of cells expressing Flag‐GBP‐1 (data not shown). Altogether, these results allowed us to exclude that p47‐GBP‐1 was produced as a result of cell death.

Caspase‐1 and Caspase‐5 are the prototypical members of a subclass of caspases involved in cytokine maturation, termed inflammatory caspases 36. Inflammatory caspases are activated by cleavage within multi‐protein complexes, termed inflammasomes, following toll‐like receptor (TLR) or P2X7 receptor engagement 35. Both caspase‐1 and caspase‐5 associate with the NALP1 inflammasome 36. The main substrates of active caspase‐1 identified to date are pro‐IL‐1β and pro‐IL‐18, the precursor forms of two related cytokines, which play critical roles in inflammation 52, 53, 54. In addition, pro‐IL‐33 and several enzymes involved in glycolysis have been shown to be targets for caspase‐1 cleavage 55, 56. Substrates of caspase‐5 are for instance the transcription factor Max and periphilin 57, 58, but caspase‐1 and caspase‐5 have also been shown to cooperate in the processing of pro‐IL‐1β 35. Secretion of pro‐IL‐1β occurs *via* a non‐classical secretion pathway involving a mechanism which has not been unravelled as yet 59. An increasing body of evidence supports the hypothesis that IL‐1β is not passively released *via* protein leakage during cell death 59, 60, 61. Actually, caspase‐1 has been shown to be generally involved in the secretion of numerous leaderless proteins, including, for instance, pro‐IL‐1α and fibroblast growth factor‐2, although those proteins are not cleaved by the protease 34. This is well in accordance with our results showing that GBP‐1 is actively secreted *via* a non‐conventional secretion pathway in a caspase‐1‐dependent manner but independently of cell death. In addition, our data showed that the cleavage and the secretion of GBP‐1 occur in endothelial cells under conditions where inflammasomes are not activated. This can be explained by the fact that caspase‐1 can also be activated by self‐dimerization of the protein, which is induced upon substrate binding 62. Recently, it has been shown that caspase‐1 dimerization can induce cleavage and subsequent secretion of pro‐IL‐1β/IL‐1β in the absence of self‐processing, and thus, in an inflammasome‐independent manner 61. Caspase‐1, like GBP‐1, belongs to the IFN‐γ‐induced genes, and its expression is increased after stimulation with the cytokine, increasing the probability of self‐dimerization 63. Hence, caspase‐1 may regulate different processes in different cells, such as inflammasome activation and IL‐1β secretion in monocytes/macrophages, or GBP‐1 cleavage and secretion in endothelial cells. Our results do not exclude that GBP‐1 may exert inflammasome‐related functions in monocytic cells, as GBP‐2 and GBP‐5 have been shown to promote NLRP3 and AIM2 inflammasome assembly in mouse and human macrophages 64, 65.

Altogether, our data support the fact that the processing and secretion of GBP‐1 by inflammatory caspases occur in IFN‐γ‐activated cells in an inflammasome‐independent manner.

## Conflict of interest statement

The authors have no conflicting financial interests to disclose.

## Grant numbers and sources of support

This work was supported by grants from the ELAN‐Fonds of the University of Erlangen‐Nuremberg to E.N. and N.B.‐L., the European Commission (FP7‐Marie Curie IEF‐221550) to N.B.‐L., the German Research Foundation [DFG: KFO257 (sub‐project 4) to M.S.; FOR 2438 (subproject 2) to E.N./M.S.; BR5196/2‐1 to N.B.L.], the emerging fields initiative (EFI) of the FAU and the W. Lutz Stiftung to M.S. and by grants to E.N./M.S. from the Interdisciplinary Center for Clinical Research (IZKF) of the Clinical Center Erlangen.

## Supporting information


**Figure S1** The monoclonal anti‐GBP‐1 antibody (clone 1B1) specifically recognizes the helical domain of GBP‐1. Hela cells were transiently transfected by Flag‐tagged GBP‐1‐helical (hel) or GBP‐1‐globular (glo) domain. Western blotting using either the 1B1 mAb or an anti‐Flag‐antibody revealed specific reaction of the 1B1 antibody with the helical domain of GBP‐1.

**Figure S2** (**A**) *In‐vitro* cleavage of GBP‐1 by caspase‐4 generates a 40‐kD fragment. Recombinant GBP‐1 (500 ng) purified from E. coli was incubated without (control) or with recombinant caspase‐1, caspase‐3 or caspase‐5 for 3 hrs at 37°C at the indicated concentrations in the absence or presence of the pan‐caspase inhibitor Z‐VAD‐fmk (Z‐VAD, 1 mM) and the caspase‐1 inhibitor Z‐YVAD‐fmk (Z‐YVAD, 0.5 mM). The reaction products were separated on a SDS‐PAGE and analyzed by Western blot using a polyclonal anti‐human GBP‐1 antibody. (**B**) Quantification of the relative amount of immunoprecipitated p47‐GBP‐1 and p67‐GBP‐1 after Z‐VAD and Z‐YVAD treatment. The band intensity of p67‐ and p47‐GBP‐1 observed on the Western‐blot depicted in Figure 2B was quantified for samples treated with IFN‐γ ± Z‐VAD or Z‐YVAD using the ImageJ software. Relative intensity is depicted in percent of the intensity observed for samples treated with IFN‐γ + 0 μM Z‐VAD/Z‐YVAD.

**Figure S3** The inflammasome is activated in THP‐1 cells by treatment with LPS/ATP. THP‐1 cells were differentiated with PMA (0.5 μM) for 3 hrs and treated with IFN‐γ (100 U/ml) as indicated. The caspase‐1 inhibitor Z‐YVAD‐fmk (Z‐YVAD, 20 μM) was added after 12 hrs. DMSO, the solvent of Z‐YVAD‐fmk, was used as negative control. LPS (1 μg/ml) and ATP (5 mM) were respectively added 6 hrs and 30 min. before harvesting of cell lysates and supernatants. Upper panel: Lysates were harvested and subjected to Western blot analysis. GAPDH was used as loading control. Lower panel: Cell culture supernatants were subjected to acetone precipitation and analyzed by Western‐blot.
