# Supplementary figures and images for: Processing and secretion of guanylate binding protein‐1 depend on inflammatory caspase activity
Source: J Cell Mol Med. 2017 Mar 8;21(9):1954–66. doi: 10.1111/jcmm.13116 (PMC5571548; doi:10.1111/jcmm.13116)

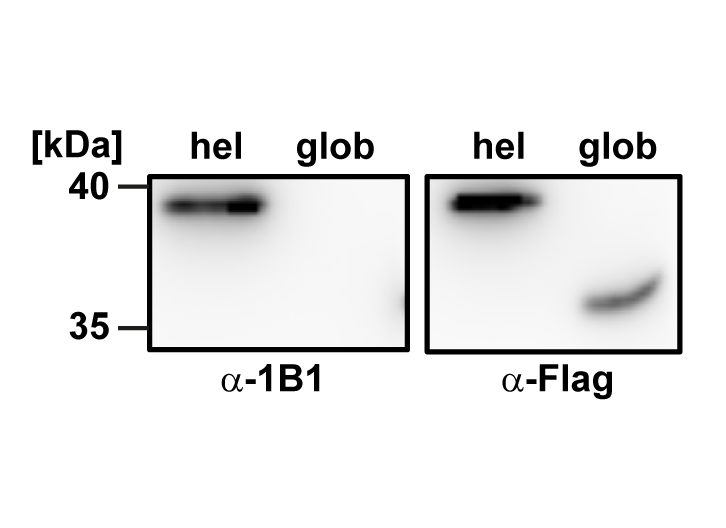

Supplement: Supplementary file 1 — Figure S1 The monoclonal anti‐GBP‐1 antibody (clone 1B1) specifically recognizes the helical domain of GBP‐1. Hela cells were transiently transfected by Flag‐tagged GBP‐1‐helical (hel) or GBP‐1‐globular (glo) domain. Western blotting using either the 1B1 mAb or an anti‐Flag‐antibody revealed specific reaction of the 1B1 antibody with the helical domain of GBP‐1. [file JCMM-21-1954-s001.tif]

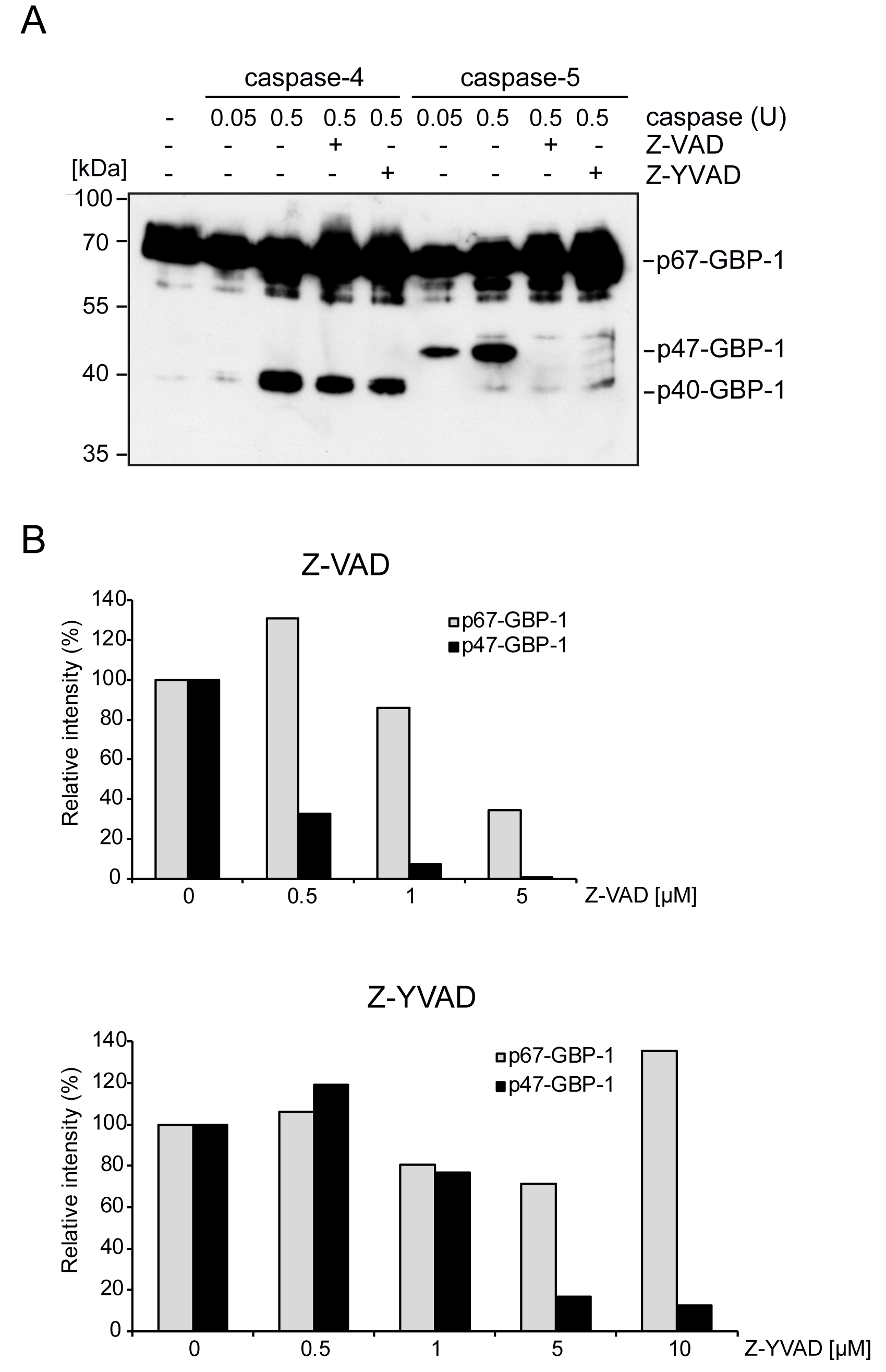

Supplement: Supplementary file 2 — Figure S2 (A) In‐vitro cleavage of GBP‐1 by caspase‐4 generates a 40‐kD fragment. Recombinant GBP‐1 (500 ng) purified from E. coli was incubated without (control) or with recombinant caspase‐1, caspase‐3 or caspase‐5 for 3 hrs at 37°C at the indicated concentrations in the absence or presence of the pan‐caspase inhibitor Z‐VAD‐fmk (Z‐VAD, 1 mM) and the caspase‐1 inhibitor Z‐YVAD‐fmk (Z‐YVAD, 0.5 mM). The reaction products were separated on a SDS‐PAGE and analyzed by Western blot using a polyclonal anti‐human GBP‐1 antibody. (B) Quantification of the relative amount of immunoprecipitated p47‐GBP‐1 and p67‐GBP‐1 after Z‐VAD and Z‐YVAD treatment. The band intensity of p67‐ and p47‐GBP‐1 observed on the Western‐blot depicted in Figure 2B was quantified for samples treated with IFN‐γ ± Z‐VAD or Z‐YVAD using the ImageJ software. Relative intensity is depicted in percent of the intensity observed for samples treated with IFN‐γ + 0 μM Z‐VAD/Z‐YVAD. [file JCMM-21-1954-s002.tif]

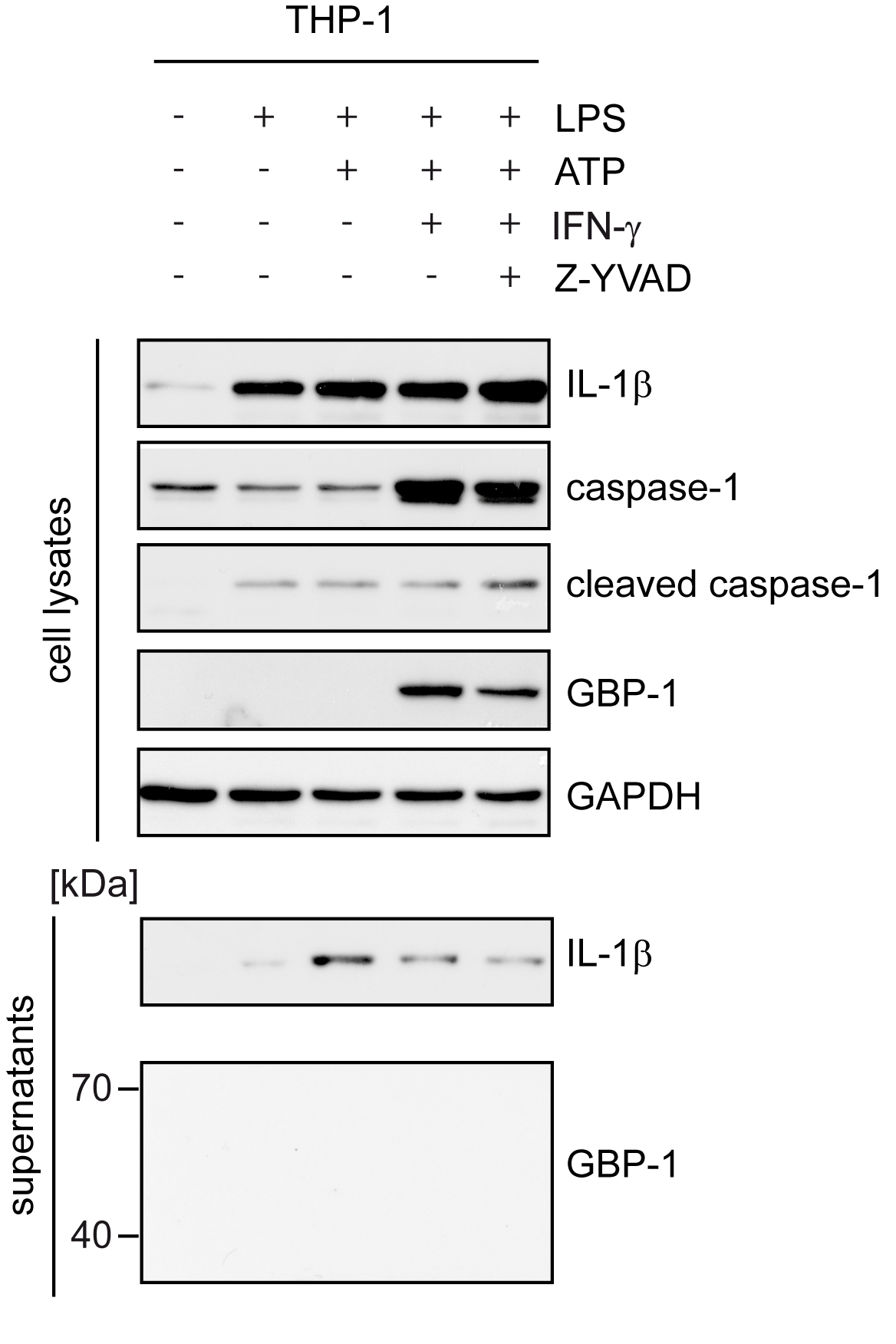

Supplement: Supplementary file 3 — Figure S3 The inflammasome is activated in THP‐1 cells by treatment with LPS/ATP. THP‐1 cells were differentiated with PMA (0.5 μM) for 3 hrs and treated with IFN‐γ (100 U/ml) as indicated. The caspase‐1 inhibitor Z‐YVAD‐fmk (Z‐YVAD, 20 μM) was added after 12 hrs. DMSO, the solvent of Z‐YVAD‐fmk, was used as negative control. LPS (1 μg/ml) and ATP (5 mM) were respectively added 6 hrs and 30 min. before harvesting of cell lysates and supernatants. Upper panel: Lysates were harvested and subjected to Western blot analysis. GAPDH was used as loading control. Lower panel: Cell culture supernatants were subjected to acetone precipitation and analyzed by Western‐blot. [file JCMM-21-1954-s003.tif]
