# Supplementary material for: Processing and secretion of guanylate binding protein‐1 depend on inflammatory caspase activity
Source: J Cell Mol Med. 2017 Mar 8;21(9):1954–66. doi: 10.1111/jcmm.13116 (PMC5571548; doi:10.1111/jcmm.13116)
Supplement: Supplementary file 4 [file JCMM-21-1954-s004.docx]

**Legends of the Supplementary Figures**

**Supplementary Figure 1: The monoclonal anti-GBP-1 antibody (clone 1B1) specifically recognizes the helical domain of GBP-1.** Hela cells were transiently transfected by Flag-tagged GBP-1-helical (hel) or GBP-1-globular (glo) domain. Western blotting using either the 1B1 mAb or an anti-Flag-antibody revealed specific reaction of the 1B1 antibody with the helical domain of GBP-1.

**Supplementary Figure 2:** **(A)** **In-vitro cleavage of GBP-1 by caspase-4 generates a 40-kDa fragment.** Recombinant GBP-1 (500 ng) purified from E. coli was incubated without (control) or with recombinant caspase-1, caspase-3 or caspase-5 for 3 h at 37°C at the indicated concentrations in the absence or presence of the pan-caspase inhibitor Z-VAD-fmk (Z-VAD, 1 mM) and the caspase-1 inhibitor Z-YVAD-fmk (Z-YVAD, 0.5 mM). The reaction products were separated on a SDS-PAGE and analyzed by Western blot using a polyclonal anti-human GBP-1 antibody. **(B)** Quantification of the relative amount of immunoprecipitated p47-GBP-1 and p67-GBP-1 after Z-VAD and Z-YVAD treatment. The band intensity of p67- and p47-GBP-1 observed on the Western-blot depicted in Figure 2B was quantified for samples treated with IFN-γ +/- Z-VAD or Z-YVAD using the ImageJ software. Relative intensity is depicted in percent of the intensity observed for samples treated with IFN-γ + 0 µM Z-VAD/Z-YVAD.

**Supplementary Figure 3: The inflammasome is activated in THP-1 cells by treatment with LPS/ATP.** THP-1 cells were differentiated with PMA (0.5 µM) for 3 h and treated with IFN-γ (100 U/ml) as indicated. The caspase-1 inhibitor Z-YVAD-fmk (Z-YVAD, 20 µM) was added after 12 h. DMSO, the solvent of Z-YVAD-fmk, was used as negative control. LPS (1 µg/ml) and ATP (5 mM) were respectively added 6 h and 30 minutes before harvesting of cell lysates and supernatants. Upper panel: Lysates were harvested and subjected to Western blot analysis. GAPDH was used as loading control. Lower panel: Cell culture supernatants were subjected to acetone precipitation and analyzed by Western-blot.
